# Evaluation of a novel device for the management of high blood pressure and shock in pregnancy in low-resource settings: study protocol for a stepped-wedge cluster-randomised controlled trial (CRADLE-3 trial)

**DOI:** 10.1186/s13063-018-2581-z

**Published:** 2018-03-27

**Authors:** Hannah L. Nathan, Kate Duhig, Nicola Vousden, Elodie Lawley, Paul T. Seed, Jane Sandall, Mrutyunjaya B. Bellad, Adrian C. Brown, Lucy C. Chappell, Shivaprasad S. Goudar, Muchabayiwa F. Gidiri, Andrew H. Shennan, the CRADLE-3 Trial Collaboration Group, Natasha L. Hezelgrave, Umesh Charantimath, Chandrappa C. Karadiguddi, Sphoorthi S. Mastiholi, Geetanjali M. Mungarwadi, Feiruz Surur, Lomi Yadeta, Yonas Guchale, Violet Mambo, Sebastian Chinkoyo, Thokozile Musonda, Christine Jere, Bellington Vwalika, Mercy Kopeka, Martina Chima, Josephine Miti, Rebecca Best, Matthew Clarke, Jesse Kamara, Jeneba Conteh, Patricia Sandi, Margaret Sesay, Fatmata Momodou, Julius Wandabwa, James Ditai, Nathan Mackayi Odeke, Annettee Nakimuli, Josaphat Byamugisha, Dorothy Namakula, Noela Kalyowa, Doreen Birungi, Emily Nakirijja, Carwyn Hill, Grace Greene, Adeline Vixama, Paul Toussaint, Grace Makonyola, Doreen Bukani, Monice Kachinjika, Jane Makwakwa

**Affiliations:** 1 Department of Women and Children’s Health, King’s College London, London, UK; 2 Department of Obstetrics and Gynaecology, KLE University’s JN Medical College, Belgaum, Karnataka India; 3 Maternity Worldwide, Community Base, 113 Queens Rd, Brighton, UK; 4 Department of Obstetrics and Gynaecology, University of Zimbabwe, Harare, Zimbabwe

**Keywords:** Stepped-wedge cluster-randomised controlled trial, Complex intervention, Vital signs, Blood pressure, Pre-eclampsia, Obstetric sepsis, Obstetric haemorrhage, Eclampsia, Maternal death, Hysterectomy

## Abstract

**Background:**

Obstetric haemorrhage, sepsis and pregnancy hypertension account for more than 50% of maternal deaths worldwide. Early detection and effective management of these conditions relies on vital signs. The Microlife® CRADLE Vital Sign Alert (VSA) is an easy-to-use, accurate device that measures blood pressure and pulse. It incorporates a traffic-light early warning system that alerts all levels of healthcare provider to the need for escalation of care in women with obstetric haemorrhage, sepsis or pregnancy hypertension, thereby aiding early recognition of haemodynamic instability and preventing maternal mortality and morbidity. The aim of the trial was to determine whether implementation of the CRADLE intervention (the Microlife® CRADLE VSA device and CRADLE training package) into routine maternity care in place of existing equipment will reduce a composite outcome of maternal mortality and morbidity in low- and middle-income country populations.

**Methods:**

The CRADLE-3 trial was a stepped-wedge cluster-randomised controlled trial of the CRADLE intervention compared to routine maternity care. Each cluster crossed from routine maternity care to the intervention at 2-monthly intervals over the course of 20 months (April 2016 to November 2017). All women identified as pregnant or within 6 weeks postpartum, presenting for maternity care in cluster catchment areas were eligible to participate. Primary outcome data (composite of maternal death, eclampsia and emergency hysterectomy per 10,000 deliveries) were collected at 10 clusters (Gokak, Belgaum, India; Harare, Zimbabwe; Ndola, Zambia; Lusaka, Zambia; Free Town, Sierra Leone; Mbale, Uganda; Kampala, Uganda; Cap Haitien, Haiti; South West, Malawi; Addis Ababa, Ethiopia). This trial was informed by the Medical Research Council guidance for complex interventions. A process evaluation was undertaken to evaluate implementation in each site and a cost-effectiveness evaluation will be undertaken.

**Discussion:**

All aspects of this protocol have been evaluated in a feasibility study, with subsequent optimisation of the intervention. This trial will demonstrate the potential impact of the CRADLE intervention on reducing maternal mortality and morbidity in low-resource settings. It is anticipated that the relatively low cost of the intervention and ease of integration into existing health systems will be of significant interest to local, national and international health policy-makers.

**Trial registration:**

ISCRTN41244132. Registered on 2 February 2016.

Prospective protocol modifications have been recorded and were communicated to the Ethics Committees and Trials Committees. The adapted Standard Protocol Items: Recommendations for Interventional Trials (SPIRIT) Checklist and the SPIRIT Checklist are attached as Additional file 1.

**Electronic supplementary material:**

The online version of this article (10.1186/s13063-018-2581-z) contains supplementary material, which is available to authorized users.

## Background

Approximately 800 women die in pregnancy or childbirth every day [1]. Obstetric haemorrhage (antepartum and postpartum), sepsis and hypertension in pregnancy account for more than 50% of maternal deaths worldwide, 99% of which occur in low- and middle-income countries (LMICs) [1]. Early detection and effective management of these conditions relies on vital sign monitoring, including pulse and blood pressure [2]. Strategies aiming to improve the detection of haemodynamic instability are vital in recognising women in need of urgent medical care.

There are simple, cost-effective and established interventions to save lives for each of these pregnancy-related conditions, but the barrier to initiating treatment lies in recognition of maternal haemodynamic compromise and access to interventions [3, 4]. Accurate blood pressure measurement is essential for detecting and monitoring pre-eclampsia, enabling antihypertensive and prophylactic anticonvulsant therapy, appropriate transfer to higher-care facilities for timed delivery and, thereby, prevention of maternal and perinatal mortality and morbidity; however, the majority of blood pressure devices that are commercially available either fail validation in pregnancy or their accuracy is unknown [5]. In LMICs, hypertension is frequently under-detected, not only because of poor availability of working and accurate blood pressure devices, but also because of inadequate training [6, 7]. Vital sign measurement is equally critical in the management of obstetric haemorrhage and sepsis [2]. Postpartum haemorrhage, the leading cause of maternal mortality in LMICs [1], can cause death within a few hours, but effective interventions are available and early recognition facilitating immediate intervention is often lifesaving [8].

The CRADLE Research Group proposes that the introduction of the CRADLE Vital Sign Alert (VSA) device, a vital-sign-measuring device and alert tool, integrated with the CRADLE training package, will aid the early recognition of haemodynamic instability secondary to haemorrhage, sepsis and pre-eclampsia in LMICs. The CRADLE VSA device has been validated as accurate for use in pregnancy, including in women with pre-eclampsia [9] and is the first device to be validated as accurate in pregnant women with low blood pressure; from example, from haemorrhage or sepsis [10]. It has been designed to be robust and longer lasting for use in community and health facility environments in LMIC settings [9, 10]. The device is affordable, easy-to-use (including for community healthcare providers), reliable and portable. The CRADLE Group has incorporated a traffic-light early warning system into the device alerting users to hypertension and shock.

This protocol describes the stepped-wedge cluster-randomised controlled trial to assess the implementation and clinical usefulness of the CRADLE intervention in LMICs.

## Methods

### Feasibility phase: November 2015 – March 2016

The trial was preceded by a mixed-methods feasibility phase. The aim was to explore the acceptability and feasibility of the CRADLE intervention and its implementation strategies in three non-trial sites representative of the 10 main trial clusters (Ramdurg, Belgaum, India, Bishoftu, Ethiopia, Masvingo, Zimbabwe). Simultaneously, the 10 main trial clusters collected primary outcome data to evaluate the methods of data collection, and to inform the randomisation programme and the sample size calculation. Results were used to optimise the final CRADLE-3 protocol including training materials and implementation strategy for the main trial.

### Definitive stepped-wedge cluster-randomised controlled trial: April 2016 – November 2017

#### Aim

The primary aim of the trial was to determine whether implementation of the CRADLE intervention to community and facility maternity care reduces a composite of (all-cause) maternal mortality or major morbidity by ≥ 25%.

The trial is complemented by simultaneous process evaluation informed by the Medical Research Council (MRC) guidance. The aim of the process evaluation is: (1) to explore if the CRADLE trial was delivered as intended (fidelity, dose and adaptation); (2) to understand whether, how and why the intervention had an impact, through exploring healthcare provider (HCP) perspectives of their usual care and of the intervention and (3) to explore if the results are likely to be scalable and sustainable. This will include an evaluation of cost-effectiveness.

#### Design and study setting

The CRADLE-3 trial was a pragmatic, mixed-methods, multicentre, stepped-wedge cluster-randomised controlled trial of the introduction of the CRADLE intervention (CRADLE VSA device and CRADLE training package) to routine maternity care settings in LMICs. It was informed by the MRC guidance for evaluation of complex interventions [11].

Ten clusters were identified to take part in the trial. Each cluster comprised a secondary or tertiary health facility with multiple satellite primary care centres that referred to the central hospital:

1. Gokak, Belgaum, India
2. Harare, Zimbabwe
3. Ndola, Zambia
4. Lusaka, Zambia
5. Free Town, Sierra Leone
6. Mbale, Uganda
7. Kampala, Uganda
8. Cap Haitien, Haiti
9. South West, Malawi
10. Addis Ababa, Ethiopia

**Fig. 1:**
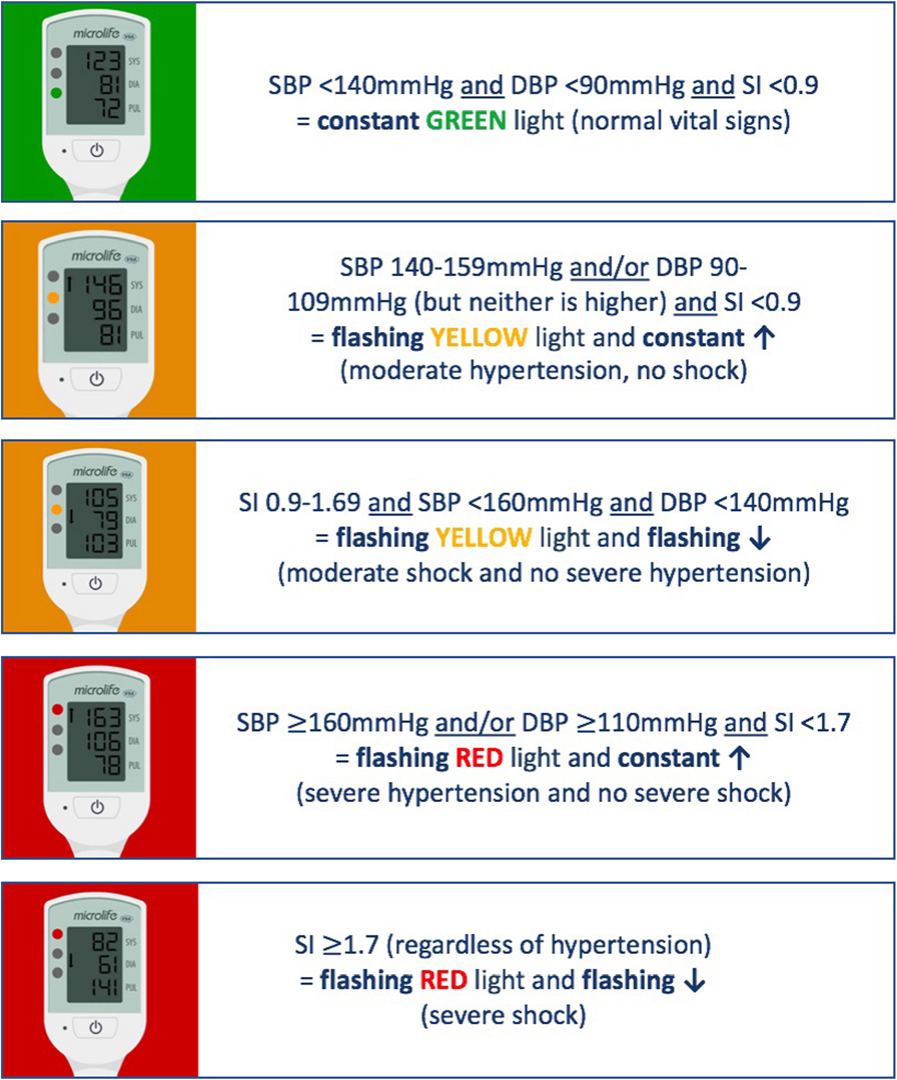
Stepped-wedge cluster-randomised controlled trial design. A schematic representation of the trial design

**Fig. 2:**
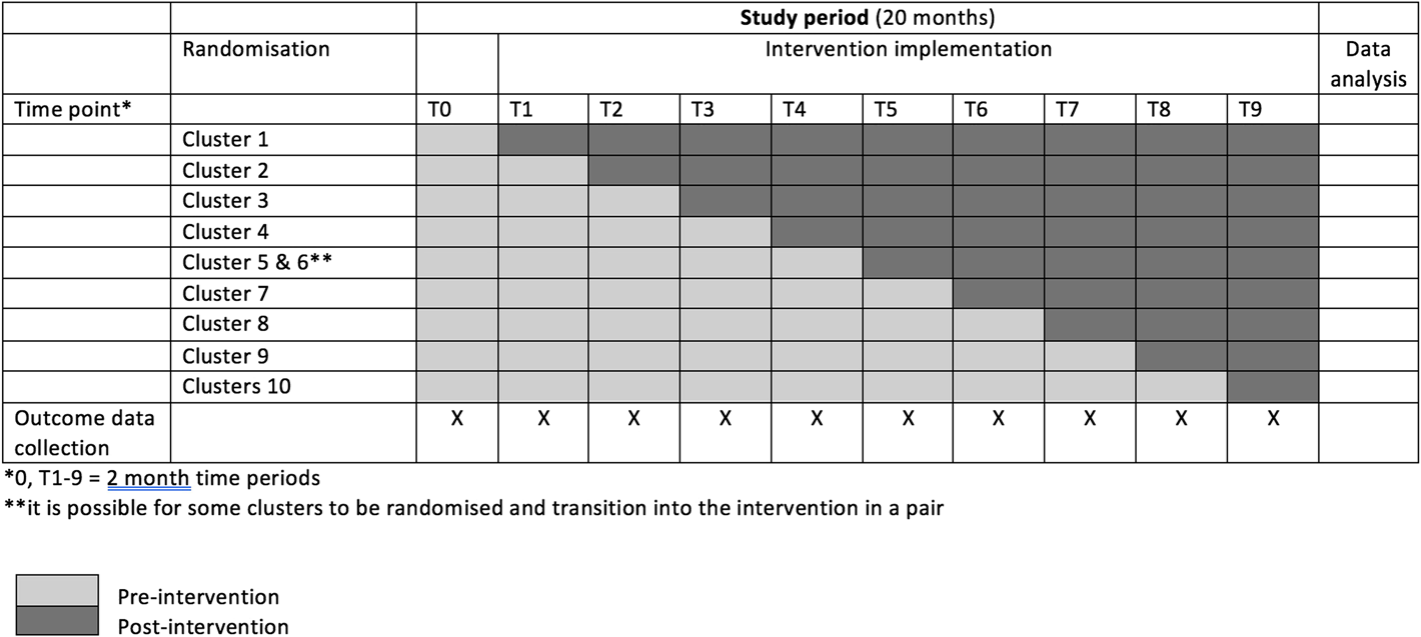
Standard Protocol Items: Recommendations for Interventional Trials (SPIRIT) Figure

Each randomisation cluster crossed over from control to the CRADLE intervention at 2-monthly intervals (Figs. 1 and 2 and Additional file 1). At the time each cluster was randomised to receive the CRADLE intervention, all levels of healthcare providers within that cluster, involved in the care of pregnant/postpartum women, had access to the CRADLE intervention. The intervention effect will be determined by comparing data points in the intervention section of the wedge with those in the controlled section.

#### Participants

##### Inclusion criteria

Women identified as pregnant or within the 6-week postpartum period, presenting for antenatal, intrapartum or postpartum care in cluster catchment areas* within the trial time frame.

*Catchment areas were defined in collaboration with local principal investigators, and included the major outreach facilities that result in women being assessed and referred to a defined central facility/ies. These were defined prior to randomisation and remained constant throughout the study period.

There were no exclusion criteria.

#### Intervention

The CRADLE intervention consisted of two components and is described according to the TIDieR Checklist [12].

#### Microlife® CRADLE Vital Sign Alert (VSA) device

The Microlife® CRADLE VSA is a hand-held, upper-arm, semi-automated device that measures blood pressure and pulse. The device has undergone extensive testing for accuracy and is one of few blood pressure devices to have been validated as accurate in pregnancy, including in pre-eclampsia and hypotension [10], as well as non-pregnant adults [13].

**Fig. 3:**
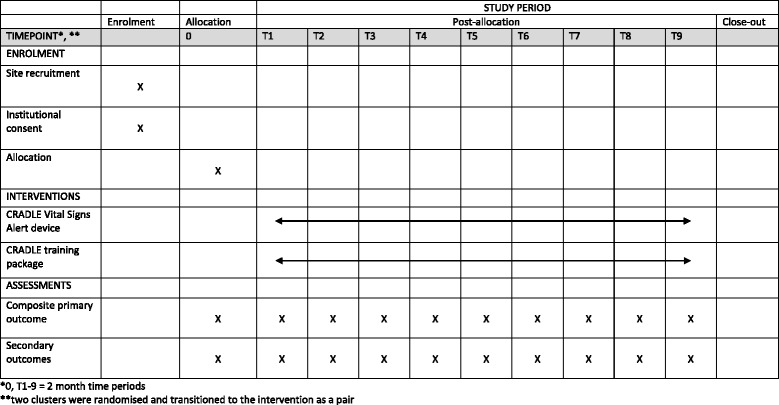
Vital Signs Alert (VSA) device traffic-light early warning system display options. Legend: *SBP* systolic blood pressure; *DBP* diastolic blood pressure; SI Shock Index

The device incorporates a traffic-light early warning system that alerts all levels of HCP to abnormalities in blood pressure and pulse secondary to obstetric haemorrhage, sepsis and pregnancy hypertension. The thresholds that trigger the traffic lights were determined through prediction studies [14, 15]. The traffic light early warning system triggers are shown in Fig. 3. A ‘red light’ and ‘up arrow’ displayed following vital signs measurement of a pregnant/postpartum woman indicate severe hypertension and should prompt intervention and/or referral. Likewise, a ‘red light’ and ‘down arrow’ with haemorrhage or sepsis should prompt immediate action. A ‘yellow light’ and either ‘down arrow’ or ‘up arrow’ indicate less urgent need for assessment, intervention and/or referral. It is proposed that the traffic lights will alert users to abnormal vital signs, including those without formal healthcare training. This should enable earlier management to improve outcomes that would directly benefit the woman and her unborn/newborn child.

The production costs are less than US$20 per unit and has been designed to be simple to use, even by unskilled personnel after minimal training. The device fulfils the World Health Organisation requirements for automated devices used in low-resource settings [13]. Other developments suited to LMICs include a micro-USB charging ability. The CRADLE VSA is manufactured in Taiwan.

As part of the CRADLE-3 trial intervention, the CRADLE VSA was incorporated into routine maternity care. Primary, secondary and tertiary facilities were allocated devices according to their delivery rate, staffing numbers and number of beds per ward. Pre-existing blood pressure measurement devices were removed from clinical areas unless existing equipment had functionality designed for that area, e.g. repeated automated measures in an operating theatre or a high dependency area, and this was left to the discretion of the lead clinician.

#### CRADLE training package

The intervention users included every cadre of healthcare professional involved in maternity care within the cluster. This included community healthcare providers (cHCPs) where they were supported at district level and involved in provision of routine maternity care. The CRADLE research group created a simple CRADLE training package for prospective CRADLE VSA users.

The training package consisted of short animated films, an interactive session, action prompt cards attached to the CRADLE VSA and posters. There were two sets of training materials available, one for facility HCP (fHCPs) and one for cHCP with very limited resources or no formal training. All materials were translated into local language where required.

The CRADLE package content covered:

- How to use the CRADLE VSA
- Maintenance of the CRADLE VSA
- Basic overview of clinical assessment and management of pre-eclampsia/eclampsia and shock in relation to the traffic-light alerts

At the randomised time point the local implementation team (clinical research officers responsible for ongoing CRADLE outcome data collection and site principal investigators) attended face-to-face one-off training with the research team lasting approximately 5 h. The implementation team and research team subsequently delivered one-off group training sessions lasting 2–4 h to local stakeholders and representative HCP from each of the clinical areas in the cluster.

Attendees were given training materials and CRADLE VSA to disseminate to their clinical areas. The implementation team continued to visit clinical areas regularly to collect outcome data therefore providing ongoing support to HCP.

The core components of the intervention (the CRADLE VSA, animated films, posters and content of the training presentation) were standardised across all clusters. Delivery of the core components could be adapted to meet the needs of the cluster.

#### Control

This intervention was compared to routine maternity care. This involved blood pressure monitoring with a variety of blood pressure devices that were available locally and management according to local guidelines.

#### Primary outcome

The rate of a composite of maternal mortality or major morbidity (one of maternal death, eclampsia or emergency hysterectomy with no double counting per 10,000 deliveries in each cluster each month). We will report the effect of the intervention on the primary endpoint, on each of the three components, and on each of the secondary endpoints.

*Maternal death* was defined as death during pregnancy or within 42 days of delivery (or last contact day if contact not maintained to 42 days).

*Eclampsia* was defined as occurrence of generalised convulsions with increased blood pressure during pregnancy, labour or within 42 days of delivery in the absence of epilepsy or another condition predisposing to convulsions.

*Emergency hysterectomy* was defined as surgical removal of all or part of the uterus.

#### Secondary outcomes

Maternal deaths from all causes were collected with additional information regarding the cause of maternal death collected to determine the potential for impact of the CRADLE VSA. The percentage of deaths that occurred as a result of obstetric haemorrhage, pregnancy-related sepsis and hypertensive disorders of pregnancy will be presented across all clusters pre and postintervention with adjusted risk ratios (RRs). If there are other large groups of other clinically important causes of death, e.g. early pregnancy complications, these will also be defined.

Maternal secondary outcome measures:

1. Intensive care unit admission, defined as any admission to a specific intensive care unit or an equivalent highest-level care environment within the trial area (or referral to the highest level care facility outside of the area) in areas where an intensive care unit does not exist
2. Stroke, defined as hemiparesis and/or blindness developed during pregnancy or in the 42 days postpartum lasting longer than 48 h
3. Cause of intensive care admission
4. Cause of maternal death
5. Cause of emergency hysterectomy
6. Place of eclamptic fit
7. Place of maternal death

##### Perinatal secondary outcomes

Number of stillbirths per 10,000 deliveries per month.

Number of neonatal deaths per 10,000 deliveries per month.

##### Process evaluation outcomes

The implementation and impact of the intervention in each site was evaluated by both quantitative and qualitative measures. The fidelity, dose, reach and adaptation (whether the intervention is implemented as planned, to the intended population and what amount) was determined by measuring:

- The duration and content of training and whether it was adapted in different contexts (structured observation of training)
- The proportion of HCPs working in maternity services that were trained to use the CRADLE device
- The proportion of facilities with access to working blood pressure devices prior to and after implementation
- The number of CRADLE VSA delivered
- The proportion of women attending maternity services that had their blood pressure measured (subset of clusters for a 4-week period immediately prior to implementation and the same duration 3 months after implementation)

The potential impact of the intervention, the acceptability and potential mechanisms of action were explored through the following methods:

- Semi-structured interviews of a purposive sample of HCPs (*n* = 3–5 in each cluster) at 3 months following implementation explored the experience of triage, intervention, referral and transportation and reception at the higher-level facility, as well as information on the escalation process and barriers to women attending higher-level care
- A focus group discussion with a purposive sample of stakeholders (*n* = 5–8 in each cluster) at 3 months elicited how the CRADLE VSA influences the process of referrals and work load
- Given our hypothesis that earlier detection will result in earlier referral and management of pregnancy complications, an evaluation of the proportion of women accessing maternity services that were referred to higher-level care was undertaken (for a 4-week period immediately prior to implementation and the same duration 3 months after implementation

The following outcomes were evaluated to explore the potential for the results to be scalable and sustainable:

- The number of CRADLE VSA reported as broken, lost or stolen (evaluated monthly after implementation to trial end; up to 18 months in the first cluster)
- The proportion of clinical areas using the CRADLE VSA post implementation (longitudinally assessed according to time of implementation up to 6, 12 and 18 months)
- Costs of implementation (including equipment, staff, meeting and training, transport)
- A focus group discussion with a purposive sample of stakeholders (*n* = 5–8 in each cluster) at 6 to 9 months and interviews with the implementation team at 12 months to elicit contextual change over time and sustainability of the intervention

Quantitative process data will be integrated into the outcome datasets to examine whether the effects of the CRADLE intervention differ by implementation. Qualitative methods will capture emerging changes in implementation, experiences of the intervention and unanticipated or complex causal pathways in addition to generating new theory about potential mechanisms of action. Quantitative and qualitative analyses will build upon one another, with qualitative data used to explain quantitative findings, and quantitative data used to test hypotheses generated by qualitative data.

The cost consequence analysis will be modelled on the basis of the equipment required and the health outcomes influenced as recorded in the trial. The incremental financial and economic cost of implementing the CRADLE intervention (device and training package) over the trial will be quantified, thereby evaluating the financial sustainability of the interventions. Research costs will be excluded from the costs of the intervention.

### Cluster description

Each cluster was described according to the number of primary, secondary and tertiary facilities and their referral distances. Details on staffing levels and availability of key resources, such as intensive care beds, blood transfusion and magnesium sulphate use, was collated. In addition, major changes to the trial catchment, such as changes to infrastructure, policy, patient payment requirements or environmental conditions, were systematically reviewed each month.

### Data collection

Methods of data collection were discussed and optimised based on the existing resources available in each site. Outcomes were triangulated across multiple sources (including referral registers, ward registers, patient records, local mortality and morbidity records and active case finding) to ensure data completeness and all outcomes checked to avoid double counting.

Outcome data were recorded over the 20-month period. Consistency and quality of source data was monitored by the research midwife/assistant at each cluster (monthly). Data were entered onto an online database (MedSciNet). This allows for extensive monitoring and query processing features, as well as a comprehensive alerting system to identify missing data. The trial coordinator will monitor data entry continuously on MedSciNet. Ten percent of the source data were validated by the primary investigator and a proportion of this was reviewed by the trial coordinator. Patient records are identified with a unique identification number generated sequentially and no identifying data are stored.

### Method of randomisation

The unit of randomisation is the cluster (or clusters), rather than individual women. Large variation in the primary event rate was anticipated between clusters; therefore, a restricted method of randomisation was used with zero rank correlation between events per month and order of randomisation. The sequence of timing for receiving the CRADLE intervention was determined by computer-generated random numbers by the CRADLE statistician. The clusters were masked to the order of implementation until they are informed of their allocation 2 months prior to their implementation date (to enable training to be arranged).

### Sample size calculation

Sample size estimation has been carried out by the CRADLE statistician, using Stata version 13.1 and the methods of Hemming and Girling [16]. This was informed by data from the feasibility phase. For the purpose of the power calculation an assumption that there are at least 4000 deliveries per centre per month was made (or 8000 per cluster period of 2 months) and at least nine clusters, each observed for 20 months (10 time periods of 2 months each). We anticipated a baseline event rate of 1% and anticipate a 25% reduction in this to 0.75% post intervention. We would require a total of 6300 outcome events, with a coefficient of variation of 0.4 and an Intra Cluster Correlation of 0.0085, to have power of 99%. We would require a total of 2450 outcome events with a coefficient of variation of 0.4, to have power of 95%.

### Statistical plan

The effect of the intervention on the primary outcome, on each of the three components, and on the secondary outcomes specified above, will be reported. Results will be reported firstly as odds ratios (ORs), with risk ratios (RRs) as a secondary comparison if the appropriate models converge. Within the stepped-wedge cluster design it remains appropriate to analyse outcome data from clusters individually, despite randomising cluster time points with some clusters paired [17].

The main statistical analysis method will be by logistic regression, using generalised estimating equations with a population-averaged model [18] (Stata command xtgee) with fixed centre effects and separate fixed linear trends in each centre for changes in outcome over time. Autoregressive correlation will account for decreasing correlations between observations over time. This model outperformed the multilevel model structure of Hussey and Hughes [19], being apparently less susceptible to bias. It gives equal weight to each woman, rather than each centre, and (being population-averaged) reports effects averaged over the length of the trial. Simulations studies suggest that using time categories, rather than linear trends do not correct well for separate time trends by centre, and can cause convergence problems. Results will be expressed as ORs; at the low event rates expected (≤ 5%), ORs and RRs are reasonably close (in the simplest case, for 5% and 3.75%, RR = 0.75, OR = 0.74). A secondary analysis to obtain RRs using a log link will be attempted, but simulation studies suggest that the convergence may be poor. We will adjust for centre effects and linear time trends and the interaction between them. This will effectively adjust for differences in baseline availability of resources between clusters.

## Discussion

We have described the protocol for a mixed-methods, multicentre, stepped-wedge cluster-randomised controlled trial. The main objective of this trial is to determine whether the introduction of the CRADLE intervention (CRADLE VSA device and CRADLE training package) to maternity care settings in LMIC communities and facilities reduces maternal mortality and morbidity. A stepped-wedge cluster-randomised controlled trial design has been chosen to evaluate the intervention in a pragmatic fashion. Individual randomisation would be logistically difficult and would not measure impact and transferability at a population level. The stepped-wedge design is useful where phased implementation is preferable because of logistical and practical constraints.

The CRADLE intervention is intended to be beneficial to all types of practice in a wide variety of settings. In keeping with the pragmatic trial design, and to ensure that results are generalisable, efforts were made to ensure that participating clusters represented diverse settings, at both a country level with different healthcare systems and at a facility level. Our study clusters, therefore, included academic/specialist facilities, private facilities and primary and secondary-level government facilities. We hypothesise that the intervention will work by facilitating earlier detection, referral and treatment of pregnancy complications.

A composite of maternal mortality or morbidity (one of maternal death, eclampsia or emergency hysterectomy with no double counting) was chosen as the primary outcome as powering a trial for maternal death alone would require a prohibitively large sample size. These are robust and meaningful clinical endpoints that are unambiguous and, therefore, feasible to collect in these environments. Maternal complications were selected, each of which is associated with significant acute and chronic morbidity. Therefore, a reduction in this composite would be highly desirable. Each component of the primary outcome will be individually interrogated to ensure no paradoxical effects within the composite, i.e. an increase in morbidity may be associated with a reduction in mortality, as a consequence of intervention. Therefore, should the CRADLE intervention result in appropriate referral and intervention (e.g. hysterectomy) this will be recognised as beneficial even though the overall effect on the composite outcome may be neutral. We have ensured that all primary outcome measures are feasible to collect at primary to tertiary facilities, as determined through the feasibility study. It is, therefore, not necessary to conduct costly household surveys. In the unlikely event that a primary outcome occurs without HCP contact, this is likely to be unchanged pre and post intervention and should not affect our ability to assess the impact of intervention implementation.

It is recognised that the CRADLE intervention may reduce perinatal mortality and morbidity. Perinatal outcomes have not been included as primary outcomes as the intervention is designed specifically to identify maternal health complications. Many of these occur postpartum and will not directly influence perinatal outcomes. Acquisition of detailed perinatal data within LMIC settings would be a substantial additional cost. However, the secondary perinatal outcomes will be collected in women who experience a primary outcome.

The potential for this trial to demonstrate reductions in maternal mortality and morbidity and a positive impact on the working lives of HCPs will be of interest to local, national and international health-policy makers. The CRADLE VSA could become a key tool in achieving the post-2015 global maternal health goals, as well as facilitating a basic recommended standard of care, i.e. accurate blood pressure determination in all pregnant women. Given the low cost, reliability and accuracy of the device, if the trial is successful, a low regulatory hurdle and rapid progression to adoption is anticipated. The trial results will be generalisable beyond the immediate research setting, as the trial will be carried out in a variety of countries and at both at community and facility levels. A robust scale-up strategy will be required to support international scale-up to allow widespread and equitable access to the innovation. This will require appropriate resourcing to include commercial expertise and facilitate partnership between inventors, manufacturers and governments to maximise effective and efficient marketing, supply and distribution through appropriate channels and fair pricing.

## Trial status

Ongoing

## Additional file

Additional file 1: SPIRIT Checklist. (DOC 122 kb)

## Abbreviations

cHCPs: Community healthcare providers
CRADLE: Community blood pressure monitoring in Rural Africa and Asia: Detection of underLying pre-Eclampsia and shock
fHCPs: Facility healthcare provider
HCP: Healthcare provider
LICs: Low-income countries
LMICs: Low- and middle-income countries
VSA: Vital Signs Alert
